# Reply to Babar and to Abu Fraiha and Leibowitz—No Place for Unprofessionalism Including Offensive Antisemitic Symbols and Regalia at Medical School Commencement Ceremonies

**DOI:** 10.5041/RMMJ.10547

**Published:** 2025-04-29

**Authors:** Steven Roth, Hedy S. Wald

**Affiliations:** 1 Michael Reese Professor of Anesthesiology and Vice Head for Research and Faculty Affairs, Department of Anesthesiology, University of Illinois College of Medicine; Professor Emeritus, University of Chicago, Chicago, Illinois, USA; 2 Clinical Professor of Family Medicine, Warren Alpert Medical School of Brown University, Department of Family Medicine, Providence, Rhode Island, USA

**Keywords:** Antisemitism, commencements, Israel-Hamas war, medical schools, professionalism, terrorism

**To the Editor**,

We thank authors Babar as well as Abu Fraiha and Leibowitz for their interest in and correspondence about our paper regarding the regalia and symbols worn by medical students and the protests and disruptions at commencement ceremonies.^1^

Babar correctly stated that “such incidents raise ethical and professional concerns, undermining the solemnity of commencement events and compromising the core principles of the medical profession, including respect, inclusivity, and impartiality.”

He cites the relevant study of Papadakis et al. showing a strong correlation between unprofessional behavior during medical training and future disciplinary actions by medical licensing boards.^2^ Students considering participation in these unprofessional activities may wish to reflect on whether they are conducting themselves in a manner that may be potentially deleterious to their future careers.

Upon review of Abu Fraiha and Leibowitz’s perspective, their approach is a reductionist one in which symbols are considered by themselves and the context in which they were presented is removed. Symbolism, however, is always vitally important in communication. It is hard to fathom how a three-part keffiyeh, Palestinian flag, and empty map of Israel with “Palestine” written adjacent in Arabic could be perceived other than representing replacement of Israel. The empty Israel map designated “Palestine” actually makes the wearer’s intention apparent as a call for Israel’s destruction.^3^

Regalia with the “Dome of the Rock” and, in Arabic, “Jerusalem is Ours,” or “Jerusalem is the capital of Palestine” present a clear message: Jerusalem does not belong to Jews, and Israel must not exist.^3^

These authors argue we should have interviewed students, apparently to learn their “intent.” But the viewer’s perception of actions and images is critically important. Every medical school requires accounting for even a perception of conflict of interest, and “microaggressions” are also not tolerated.^4^ It appears that the authors hold students to a different standard when wearing potentially offensive regalia, which can be perceived as an effort to excuse this offensive, unprofessional behavior.

Yasser Arafat converted the keffiyeh into a terrorist symbol by donning it in the exact shape of Israel.^5^ Today it is worn by Ayatollahs and terror groups Hamas and Hezbollah, among others. The keffiyeh is not neutral when connected to anti-Israel protests.^6^ It is now associated with violence against and is offensive to Jews as supporting terror. Even if deemed “controversial,” and conveying implicit if not explicit offensive meanings, professionalism mandates sensitivity to not don it during such a hallowed occasion.

The authors suggest use of an alternative definition of antisemitism without compelling rationale. The International Holocaust Remembrance Alliance (IHRA) definition is the most widely accepted and recognized one and, as such, is the major tool for identifying contemporary antisemitism.^7^ As of February 1, 2025, 1,266 entities worldwide adopted the definition, encompassing 45 countries including the USA, 37 US states, and 98 US city and county governments.^8^ Abu Fraiha and Leibowitz’s own state, and their employer, Harvard, have accepted the IHRA as the official definition.

The authors state that we “mistakenly conflate antisemitism with harsh criticism of the Israeli government and the actions of its military as well as legitimate acts of solidarity with people under oppression.” Criticisms of governmental policy are not equivalent to the antisemitic acts discussed in our paper. False equivalences of these acts with “acts of solidarity with people under oppression” are unhelpful. In their words, “physicians engaged in their surrounding society cannot remain silent amidst horrific human-made humanitarian catastrophes, including the one that has been unfolding in Gaza over the past 15 months.” We agree. If students caring so deeply about humanitarianism are led on this unique day to choose to demonstrate, contravene professionalism, and disrupt their own commencements, the absence of concomitant protests against Hamas atrocities is conspicuous. Hamas’s barbaric actions including murders of children as young as 9 months, weaponization of hospitals, kidnapping of Israelis and citizens of over 10 different countries, rapes, doctors holding hostages for Hamas, and Hamas’s appropriation of supplies intended for civilians led directly to the catastrophe that has unfolded in Gaza.^9^

In regard to the authors’ raising the issue of our not presenting rules for commencement, explicit rules are only occasionally found on commencement websites, as we had noted. Every medical school, however, has rules concerning what contravenes an Accreditation Council for Graduate Medical Education (USA) required competency, professionalism. Stanford School of Medicine, for example, clearly delineates its elements:

Professionalism extends beyond interactions with patients and their families … [it] … also involves relationships and interactions between all those involved in medical education and the delivery of patient care including physicians, students, administrators, and allied health professionals. The elements of professionalism include altruism, accountability, responsibility, excellence, duty, honesty, integrity, and respect for others.^10^

Indeed, if we, as the authors claim, “educate our trainees to consider all available empirical evidence, to be sensitive to patients’ cultural background, and to be attuned to what they might perceive as injustices,” we would certainly expect proper judgment from a graduating medical student for what is disruptive, insensitive, and offensive. We cannot help but wonder how a solemn ceremony could possibly be construed as the place for these behaviors.

We can only imagine how offensive, insensitive, and frightening it was to graduating students, the audience, faculty, and others viewing when their very own Harvard Medical School graduate displayed a white coat with red (blood) palm prints with which she came up onto the stage.^1^ This deplorable choice is reminiscent of the bloody palms infamously displayed by the terrorist murderers of Jews who inadvertently wandered into Ramallah in 2000 and which was reported around the world by nearly every major media outlet.^11^ Displaying signs conveying false charges about Israel bombing “hospitals” (which lost neutrality when they became bases of Hamas terrorists)^12^ and accusing Israel of genocide (contrary to the definition, but also precisely Hamas’s stated goal for Israel)^13^ as well as wearing boycott, divest, and sanction buttons are all antisemitic acts. Some students even called for an “end to Zionism,” a denial of the Jewish right to self-determination in their ancient homeland, offensive in its blatant antisemitism.

Abu Fraiha and Leibowitz tout the need for students to “express outrage at loss of life in Gaza,” yet we note their abject, unacceptable failure to address the source as Hamas which harmed Gazan civilians through terrorism and attacked Israel leading to the existential threat to Israel’s survival. All humanity should be outraged at the loss of life in Gaza and Israel as well as barbaric Hamas cruelty dedicated to destruction of Israel and death of all Jews.^3^ Abu Fraiha and Leibowitz’s omission of this pertinent context resonates with that seen at the commencements. In regard to these authors’ singular focus, we note their and the medical students’ “unjustified silence” regarding the war in Congo,^14^ Sudan,^15^ and other major ongoing geopolitical conflicts with dire circumstances for civilians, which apparently were not deemed a priority for commencement displays, protests, and disruptions.

The behaviors at commencement represent a highly public demonstration of the widespread antisemitism in academic medical centers of the US and other Western countries since October 7, 2023.^16^–^18^ As this offensive, insensitive, unprofessional, and antisemitic behavior of US medical students, supported by some faculty,^19^ becomes publicly exposed, Americans’ compromised trust in physicians can be expected to worsen. Indeed, a governmental task force (US Departments of Justice, Education, and Health and Human Services) has initiated an investigation into, and consideration of withholding federal grant funds from medical schools (including Harvard) for this antisemitic harassment.^20^

Abu Fraiha and Leibowitz sanctimoniously labeled our work “biased.” However; their own bias is overtly presented within unfounded allegations, i.e. “… delegitimizing critique on the rights-abusive policies and war crimes of the Israeli government.” To conclude, concerning their title “There Should Be a Clear Distinction Between Legitimate Protest and Antisemitism,” medical school commencements are not the place nor time for protest, hatred, and support for terror. Rather, it is a celebration of significant achievement, a time to express gratitude to faculty, medical school staff, and their fellow students for the education they received, and the time to take a sacred oath of professionalism and duty. Our peer-reviewed findings have clear meanings substantiated by resulting federal probes of medical schools for antisemitic harassment, of which this behavior was just one very public manifestation since October 7, 2023.^20^ Enforcement of policies for non-hostile learning and practice environments and upholding of professionalism standards in regard to Jewish students and staff is urgently needed as well as policies for protecting all patients from hatred, harassment, and discrimination.^16^

## Abbreviations

IHRA: International Holocaust Remembrance Alliance
